# An Ensemble Classifier to Predict Protein–Protein Interactions by Combining PSSM-based Evolutionary Information with Local Binary Pattern Model

**DOI:** 10.3390/ijms20143511

**Published:** 2019-07-17

**Authors:** Yang Li, Li-Ping Li, Lei Wang, Chang-Qing Yu, Zheng Wang, Zhu-Hong You

**Affiliations:** 1 School of Information Engineering, Xijing University, Xi’an 710123, China; 2 College of Information Science and Engineering, Zaozhuang University, Zaozhuang 277100, China

**Keywords:** protein–protein interactions, position-specific scoring matrix, rotation forest, protein sequence

## Abstract

Protein plays a critical role in the regulation of biological cell functions. Among them, whether proteins interact with each other has become a fundamental problem, because proteins usually perform their functions by interacting with other proteins. Although a large amount of protein–protein interactions (PPIs) data has been produced by high-throughput biotechnology, the disadvantage of biological experimental technique is time-consuming and costly. Thus, computational methods for predicting protein interactions have become a research hot spot. In this research, we propose an efficient computational method that combines Rotation Forest (RF) classifier with Local Binary Pattern (LBP) feature extraction method to predict PPIs from the perspective of Position-Specific Scoring Matrix (PSSM). The proposed method has achieved superior performance in predicting *Yeast*, *Human*, and *H. pylori* datasets with average accuracies of 92.12%, 96.21%, and 86.59%, respectively. In addition, we also evaluated the performance of the proposed method on the four independent datasets of *C. elegans*, *H. pylori*, *H. sapiens*, and *M. musculus* datasets. These obtained experimental results fully prove that our model has good feasibility and robustness in predicting PPIs.

## 1. Introduction

Protein is the essential part of the life activities of cells and organisms [1], and its function is usually performed by interacting with other proteins [2]. With the development of high-throughput biotechnology, experimental methods such as mass spectrometry, microarray analysis, and *Yeast* two-hybrid system have been widely used to detect protein–protein interactions (PPIs) [3,4,5,6,7,8]. However, these biological experimental methods are not only expensive and time-consuming, but also have a high false positive rate. In addition, the experimentally identified PPI can only cover a small portion of the entire PPIS network. Therefore, it is particularly important to design an accurate and effective computational method to predict PPIs.

At present, many computational methods have been proposed for predicting PPIs. These methods are usually based on the information of gene co-expression, phylogenetic relationship, and three-dimensional structural and so on [9,10,11,12,13,14,15,16,17,18,19,20,21]. Although these methods have achieved excellent results, they need to rely on prior knowledge of proteins [22]. Therefore, in order to overcome this drawback, many researchers have proposed the PPIs prediction method based on protein amino acid sequence information in recent years [23,24,25,26,27,28]. This kind of method can use the machine learning algorithm to extract important information from protein sequence data, and extract key features through feature extraction methods, so as to accurately and effectively predict the relationship among proteins [29]. For example, Shen et al. [30] rely on the properties of amino acids to extract the features of protein sequences by adopting the method of the conjoint triad. In order to reduce the dimension of the feature vector space, they divide 20 amino acids into 7 groups, which is determined by the volume of the side chain and the dipole. Zhou and Yang [31] separated the entire protein sequence into different local regions of different lengths, and then obtained three local descriptors of each local region, so as to further study the overlapping continuous and discontinuous interactions in the protein sequence [32]. Nakashima et al. [33] used the method of amino acid composition (AAC) to detect PPIs. The final experimental results show that this method can effectively predict PPIs. Guo et al. [34] proposed a combination of auto covariance (AC) and support vector machine (SVM) to predict PPIs. AC can efficiently obtain the interaction between a certain number of amino acids and amino acids in the sequence. Under the classification of SVM, the model achieved 87.36% accuracy on *Yeast* dataset. Zhou et al. [32] used a combination of local descriptors (LD) and support vector machines (SVM) to predict PPIs. The model achieved a prediction accuracy of 88.56% on the *Yeast* dataset. Wang et al. presented a computational model called PCVMZM to detect PPIs from protein amino acid sequences based on Zernike moments descriptor and probabilistic classification vector machines. This method yielded excellent performance on the *Yeast* dataset, and an average prediction accuracy of 94.48% indicates that the method is reliable for predicting protein–protein interactions.

In this study, we propose a novel sequence-based method to predict protein–protein interactions by combining Local Binary Pattern (LBP) feature extraction method and Rotation Forest (RF) classifier. More specifically, the method first converts the protein sequence information into a numerically represented Position-Specific Scoring Matrix (PSSM), then uses LBP to extract the effective features of the protein, and finally sends them into the RF classifier for accurate prediction. In the experiment, we used PPIs datasets of *Yeast*, *Human*, and *H. pylori* to evaluate the performance of the proposed model. The evaluation results show that our model achieved an average accuracy of 92.12%, 96.21%, and 86.59% on the three datasets, respectively. For the sake of verifying the reliability of our method, we have also predicted the protein–protein interactions on four independent datasets of *C. elegans, H. pylori, H. sapiens*, and *M. musculus* datasets and their accuracies are 94.82%, 94.79%, 95.11%, and 93.93%, respectively.

## 2. Results and Discussion

### 2.1. Performance Evaluation

**Figure 1:**
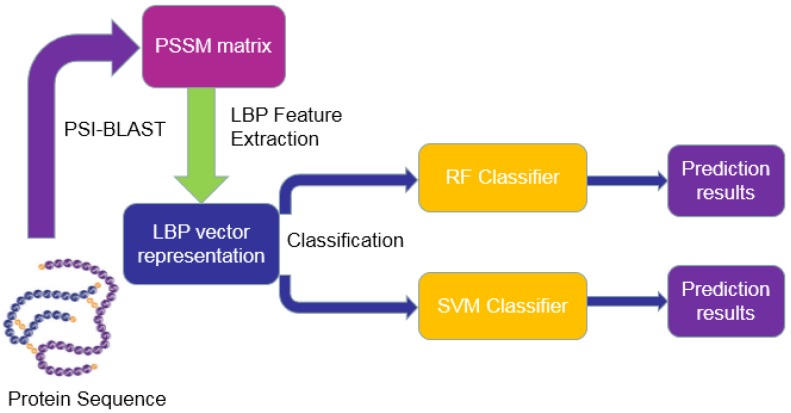
The workflow of the proposed method.

To make the experimental results more reliable, we implemented the 5-fold cross-validation on all data to evaluate the performance of the proposed method. The evaluation index of the model includes overall prediction accuracy (ACC), sensitivity (SN), precision (PE), and Matthews correlation coefficient (MCC). The calculation formula for the evaluation criteria are as follows:

(1) $$ACC=\frac{T_{P}+T_{N}}{T_{P}+T_{N}+F_{P}+F_{N}}$$

(2) $$SN=\frac{T_{P}}{T_{P}+F_{N}}$$

(3) $$PE=\frac{T_{P}}{T_{P}+F_{P}}$$

(4) $$MCC=\frac{T_{P}\times T_{N}-F_{P}\times F_{N}}{\sqrt{\left(T_{P}+F_{P})\times (T_{P}+F_{N})\times (T_{N}+F_{P})\times (T_{N}+F_{N}\right)}}$$

where True Positive $\left(T_{P}\right)$ indicates the number of positive samples that are correctly predicted. False Positive $\left(F_{P}\right)$ refers to the number of positive samples that are incorrectly predicted. True Negative $\left(T_{N}\right)$ indicates the number of negative samples that are correctly predicted. False Negative $\left(F_{N}\right)$ represents the number of negative samples that are incorrectly predicted. At the same time, the Receiver Operating Characteristic (ROC) curves and the Area Under a Curve (AUC) are also used as an evaluation index to assess the performance of the model [35]. The workflow of the proposed model is shown in Figure 1.

### 2.2. Assessment of Prediction Ability

In order to obtain more accurate and reliable experimental results, we optimized two important parameters of the rotation forest classifier on three different datasets of *Yeast*, *Human*, and *H. pylori*. Through the grid search method, we get the number of the optimal feature subset K of RF classifier is 10, and the number of the optimal decision trees *L* is 21. Meanwhile, we utilized a 5-fold cross-validation method to avoid over-fitting of the results. Specifically, we divide the total dataset into five roughly equal subsets, four of which are used as a training set and the rest one as a test set. This process is executed five times until all subsets are used as a test set once and only once. Finally, we take the average and standard deviation of the five experiments as the experimental results of the model. The prediction results of the three datasets are shown in Table 1. Additional materials are available online, Tables S1–S3.

**Figure 2:**
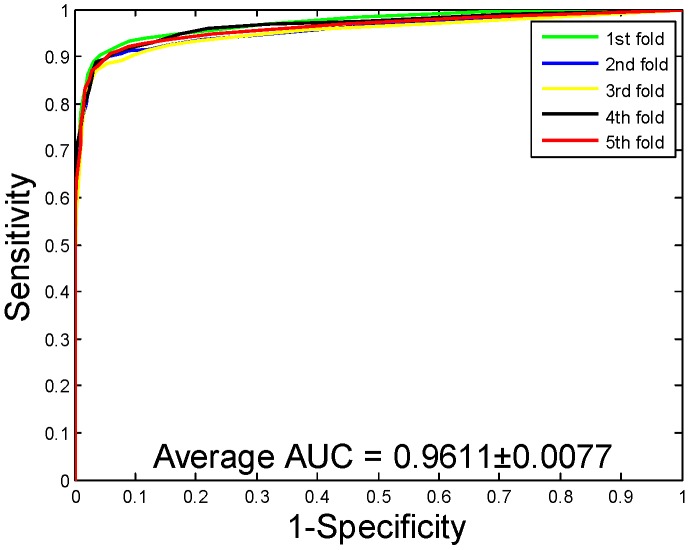
Receiver Operating Characteristic (ROC) curves are performed by the proposed method on *Yeast* protein–protein interactions (PPIs) dataset.

**Figure 3:**
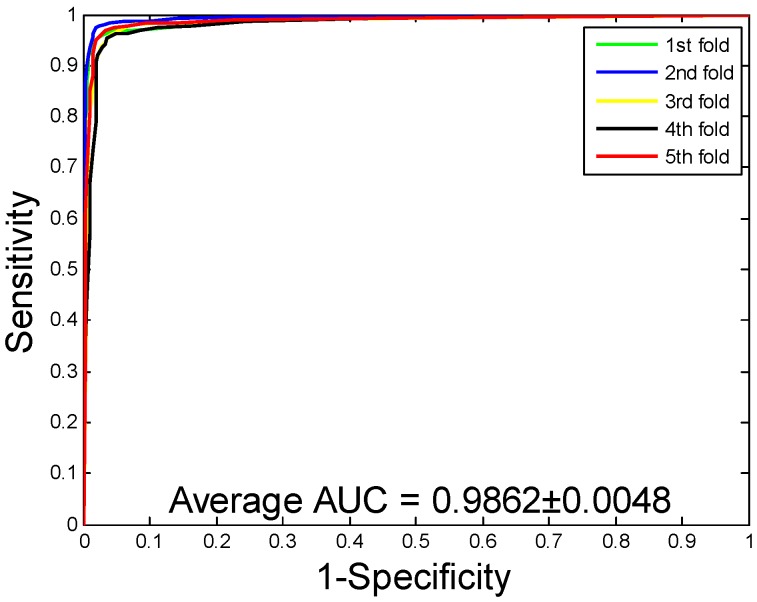
Receiver Operating Characteristic (ROC) curves are performed by the proposed method on *Human* protein–protein interactions (PPIs) dataset.

**Figure 4:**
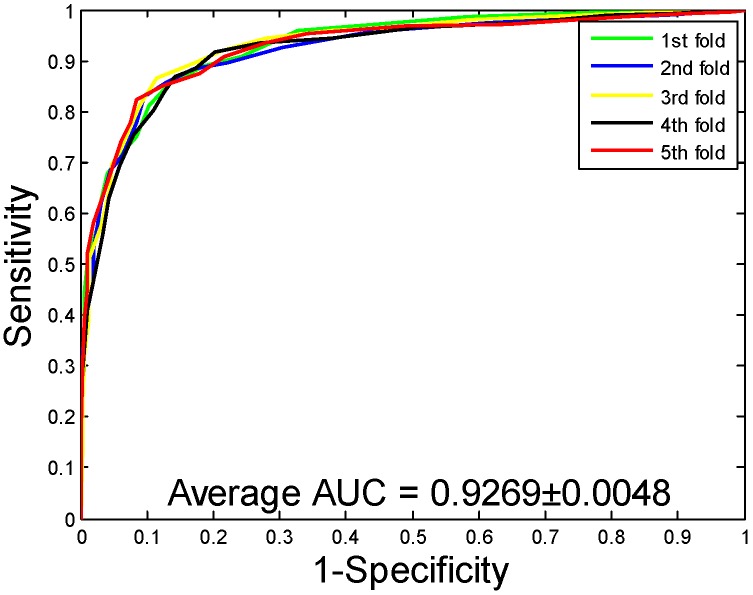
Receiver Operating Characteristic (ROC) curves are performed by the proposed method on *H. pylori* protein–protein interactions (PPIs) dataset.

When our method is used to predict the PPIs of the *Yeast* dataset, the average accuracy, precision, sensitivity, and MCC of the prediction results are well displayed, which are 92.12%, 94.20%, 89.76%, and 85.46%, respectively. The standard deviations of these predicted results are 0.54%, 0.78%, 0.96%, and 0.92%, respectively. When our method is adopted to predict the PPIs of the *Human* dataset, our method also obtains good prediction results of average accuracy, precision, sensitivity, and MCC, which are 96.21%, 97.23%, 94.77%, and 92.70%, respectively. The standard deviations of these predicted results are 0.76%, 1.19%, 1.09%, and 1.42%, respectively. When our method was utilized to predict the PPIs of the *H. pylori* dataset, the average accuracy, precision, sensitivity, and MCC were predicted to be 86.59%, 87.70%, 85.17%, and 76.73%, respectively. The standard deviations of these predicted results are 0.48%, 1.89%, 2.20%, and 0.74%, respectively. The ROC curves of the proposed model on three datasets are Figure 2, Figure 3 and Figure 4. Here, the X-axis indicates the false positive rate, while the Y-axis denotes the true positive rate. In order to better verify the feasibility of our method, AUC values are calculated on *Yeast*, *Human*, and *H. pylori* datasets and their average AUC values are 96.11%, 98.62%, and 92.69%, respectively.

### 2.3. Comparison with Support Vector Machine (SVM) Classifier

To more clearly assess the impact of the RF classifier on model performance, we compare the results of RF classifier model with those of Support Vector Machine (SVM) classifier model on the same dataset. To be fair, the data fed into the two classifier models are identical, both of which have undergone numerical transformation and feature extraction. The LIBSVM tool package used by SVM can be downloaded from its official website https://www.csie.ntu.edu.tw/~cjlin/libsvm/. When using SVM, the regularization parameter *c* and the kernel parameter *g* are optimized by taking a grid search method. Eventually, we set *c* as 10 and *g* as 60 on the *Yeast*, *Human*, and *H. pylori* datasets, respectively.

**Figure 5:**
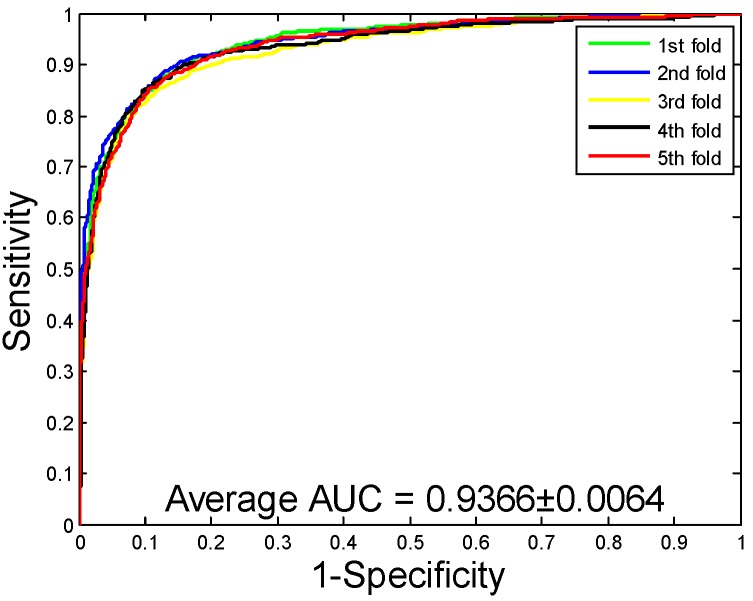
Receiver Operating Characteristics (ROC) curves are performed by the Support Vector Machine (SVM) method on *Yeast* protein–protein interactions (PPIs) dataset.

**Figure 6:**
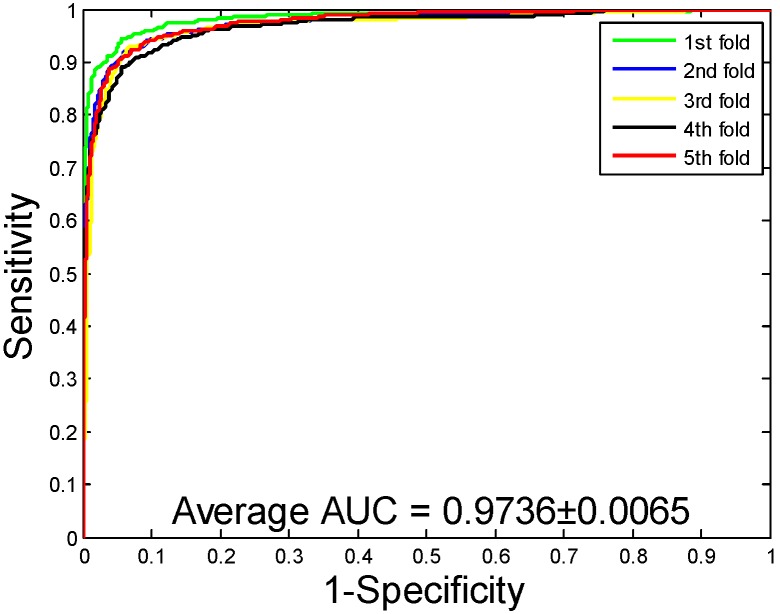
Receiver Operating Characteristics (ROC) curves are performed by the Support Vector Machine (SVM) method on *Human* protein–protein interactions (PPIs) dataset.

**Figure 7:**
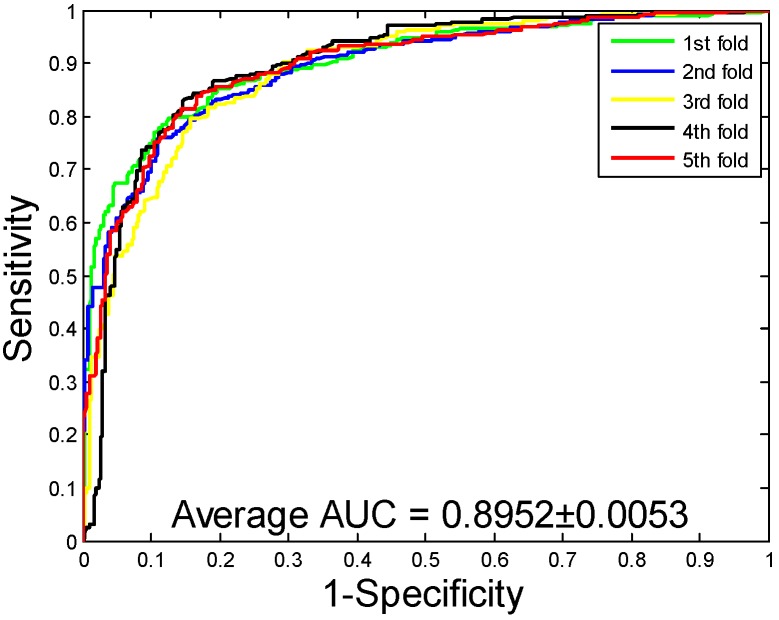
Receiver Operating Characteristics (ROC) curves are performed by the Support Vector Machine (SVM) method on *H. pylori* protein–protein interactions (PPIs) dataset.

The experimental results generated by the proposed model and the SVM model on the three datasets are summarized in Table 2. From the table, we can see that the average accuracy, precision, sensitivity, and MCC of the SVM model generated on the *Yeast* dataset are 86.99%, 88.05%, 85.62%, and 77.36%, respectively. When exploring the PPIs of the *Human* dataset through SVM model, the average accuracy, precision, sensitivity, and MCC obtained are 92.56%, 93.71%, 90.47%, and 86.18%, respectively. When the SVM is used to predict the PPIs of *H. pylori* dataset, the average accuracy is 81.62%. By comparing the results of two classifier models on the three datasets, we can see that the accuracy of the classifier based on SVM is lower than that of RF classifier. The results of the ROC curves on the three datasets predicted by the SVM classifier are reflected in Figure 5, Figure 6 and Figure 7. Through observing and analyzing the results in the table, we can see that the model based on RF classifier has better performance than SVM classifier model in predicting PPIs.

### 2.4. Comparison with Existing Methods

In order to better evaluate the performance of the proposed method, we compare it with other existing methods on the same dataset. Table 3 and Table 4 show the results obtained by different methods on the *Yeast* and *Human* datasets. As can be seen from Table 3, there are six methods applied to the *Yeast* dataset. Among them, our method shows a good average accuracy, which is 92.12%. In addition, the standard deviation obtained by the proposed model is also low. It can be seen from Table 4 that the proposed method also achieves better overall performance on the *Human* dataset. These results indicate that the proposed model has better performance and robustness than other methods on the *Yeast* and *Human* dataset.

There are two main reasons for this result: The first is that we use a sequence-based approach to predict PPIs. The discriminative information contained in the protein sequence combined with the effective LBP feature extraction method can contribute to the improvement of model performance. The second is that we use the ensemble classifier RF, which can synthesize the results of each sub-classifier and effectively improve the accuracy of prediction.

### 2.5. Performance on Independent Datasets

By analyzing the results obtained from previous experiments, it is no exaggeration to say that our method gives superior performance in predicting PPIs on three datasets. In this part of the experiment, we validated the performance of the proposed method using an independent dataset, which were selected from the Database of Interacting Proteins(DIP) database, namely *C. elegans*, *H. pylori*, *H. sapiens*, and *M. musculus* datasets. In the experiment, we train the model with all of the 11,188 protein pairs in the *Yeast* dataset, and then predict the PPIs of the four independent datasets. The experimental results are listed in Table 5. From table we can see that the accuracies of the proposed model on *C. elegans*, *H. pylori*, *H. sapiens*, and *M. musculus* datasets were 94.82%, 94.79%, 95.11%, and 93.93%, respectively. The proposed model achieves high accuracy in all four independent datasets, which indicates that the proposed model has strong competitiveness in predicting the PPIs of different species.

## 3. Materials and Methodology

### 3.1. Dataset and Data Collection

In this paper, we employed a highly credible PPIs dataset of *Saccharomyces cerevisiae*, which comes from the open Database of Interacting Proteins (DIP) [38]. Since this dataset contains a large number of homologous proteins, in order to eliminate the differences, we deleted more than 40% sequence identities in these homologous sequences. At the same time, lower than 50 residues of protein pairs will also be removed, because they may be only a small fragment. After this treatment, the remaining 5594 protein pairs are established, which are used as positive datasets. In addition, 5594 additional protein pairs in different subcellular localization are also constructed, which are considered as negative datasets [12]. Eventually, we built a total *Yeast* dataset consisting of 11,188 protein pairs, half of which came from the positive dataset, and the other half from the negative dataset. Similarly, we constructed *Human* and *Helicobacter pylori* (*H. pylori*) datasets. The *Human* dataset contains 8161 protein pairs, of which 4262 negative protein pairs were used to construct the negative dataset and 3899 positive protein pairs were used to construct the positive dataset. The *H. pylori* dataset contains 2916 protein pairs, half of which are positive datasets and the other half are negative datasets.

### 3.2. Position-Specific Scoring Matrix (PSSM)

Protein sequences have undergone various changes in the process of biological evolution. With these constant changes, one or more amino acid residues are displaced, inserted, or deleted in the protein sequence, and the comparability between proteins has also decreased gradually. However, these homologous proteins may still have similar structures. Therefore, in order to demonstrate this characteristic of proteins, we introduce the Position-Specific Scoring Matrix (PSSM) which can fully acquire the evolutionary information of protein sequences. In the experiment, we make use of the Position-Specific Iterated BLAST (PSI-BLAST) search tool to generate PSSMs on the local machine [39]. In order to obtain reliable homologous sequence data, we optimize its main parameters, in which E-value is set to 0.001 and the number of interactions is set to 3, respectively. PSI-BLAST toolkit can be downloaded from http://blast.ncbi.nlm.nih.gov/Blast.cgi. PSI-BLAST will return a PSSM where each PSSM is R rows and 20 columns. The PSSM can be defined as:

(5) $$\mathrm{PSSM}=\left[\begin{array}{cccc} \rho_{1,1} & \rho_{1,2} & \cdots & \rho_{1,20}\\ \rho_{2,1} & \rho_{2,2} & \cdots & \rho_{2,20}\\ \vdots & \vdots & \vdots & \vdots \\ \rho_{R,1} & \rho_{R,2} & \cdots & \rho_{R,20} \end{array}\right],$$

where R expresses the length of the amino acid sequence and 20 stands for 20 amino acids. The value of $\rho_{i,j}$ in the PSSM indicates that the ith amino acid residue is mutated into the type j amino acids among the 20 native amino acids.

### 3.3. Local Binary Pattern (LBP)

Local Binary Pattern (LBP) is an effective algorithm for describing the local texture features of an image [40]. It has significant features of rotation invariance and grayscale invariance. At present, LBP has been widely used in image processing, including facial expression recognition, image recovery and scene analysis [41,42]. The original LBP operator is defined as the window of 3×3, which uses the gray value in the fixed neighborhood. As a result, the texture information around the image pixels is unlikely to be obtained correctly. Ojala et al. [43] proposed the original LBP operator, which uses the central pixel value of the window as a threshold and gives the 8-bit codes through the eight pixel values around the center pixel. For the sake of adapting the texture features of different scales, researchers improved the original LBP operator, in which the operator was extended to any radius and neighborhood, while the original square neighborhood was replaced with a circular neighborhood. The LBP operator can have any number of pixels in a circular neighborhood of radius R. Therefore, the circular LBP operator with radius R can be obtained.

In this experiment, LBP features of all PSSM matrices can be calculated. Where N is used to indicate the number of neighboring pixels around the center pixel, and R is employed to represent the radius of a circle around N equidistant neighborhoods of the center pixel. Here, we set the corresponding parameters of the LBP. R=1 represents a circular neighborhood with a radius of 1, and N=8 represents an LBP operator with eight sample points in the circular neighborhood. The $i_{c}$ is used to represent the luminance value of the center pixel and $i_{i}$ to represent the intensity value of the circular neighborhood. The central pixel is regarded as the threshold of the window, and then the gray values of the eight neighboring pixels are compared with them. If the surrounding pixel value is greater than the central pixel value, its pixel position is marked as 1. Otherwise, it is marked as 0. The formula calculation of Local Binary Pattern can be defined as follows:

(6) $$B=b\left(s\left(i_{0}-i_{c}\right),s\left(i_{1}-i_{c}\right),\ldots ,s\left(i_{N-1}-i_{c}\right)\right),$$

where

(7) $$s(x)=\{\begin{array}{c} 1\\ 0 \end{array}\begin{array}{c} ,x\geq 0\\ ,x<0 \end{array}$$

(8) $$LBP_{N,R}(x_{c},y_{c})=\sum_{i=0}^{N-1}s(i_{i}-i_{c})2^{i}$$

here, the appropriate gray value in a circular neighborhood is calculated as [43].

(9) $$i_{i}=I\left(x+R\sin\frac{2\pi i}{N},y-R\cos\frac{2\pi i}{N}\right)$$

where $\left(x_{c},y_{c}\right)$ represents the gray value $i_{c}$ of the center pixel in the LBP. The rotation invariance problem can be solved by selecting the smallest binary number of all LBPs. There are 256 kinds of LBP features in the experiment when all possible outcomes are considered among neighborhoods. Finally, we extract the LBP features of PSSM, each of which is the feature matrix of the 1×256.

### 3.4. Rotation Forest (RF)

Rotation forest (RF) is an ensemble classifier consisting of a set of decision trees. It was proposed by Rodriguez et al. [44]. For each decision tree in the RF, the bootstrap sample is derived from the original training set to be used to form a new training set. The feature set of the new training set is randomly divided into several subsets and transformed using a linear transformation method. Thus, a complete feature set can be reconstructed by transforming all the features of each tree during the ensemble process. Since a small rotation of axis can construct completely different trees, the transformation method can guarantee the diversity of the ensemble system. Finally, we can use the main voting rules to fuse the output of all trees.

Let the training sample set X be an N×n matrix, which contains N training samples and n features. Let F be the feature set and the corresponding label vector be $Y={\left(y_{1},y_{2},\ldots ,y_{n}\right)}^{T}$ with size N×1. Suppose that the feature set of the sample set is randomly partitioned into K subsets with the same size. In this case, the decision tree L in the RF can be represented as $T_{1},\ldots ,T_{L},$ respectively. Here, we need to determine the two parameters L and K in advance. The implementation of the rotation forest classifier is as follows:

(1) The feature set F is randomly divided into K disjoint subsets, and each subset contains M=n/K features.

(2) Assuming that $F_{ij}$ be the *j*th subset of features, which is used to train the classifier $T_{i}.$ Let $X_{ij}$ be the dataset for X. For each subset, a nonempty random subset is selected for $X_{ij}.$ Then, a bootstrap resampling is selected from $X_{ij}$ with a size of 75% of the dataset to generate a new training set $X_{ij}^{\prime }.$

(3) Apply principal component analysis to $X_{ij}^{\prime }$ to produce the coefficients in matrix $C_{ij}.$ The size of each $X_{ij}^{\prime }$ is M×1 with the coefficients of $\lambda_{ij}^{\left(1\right)},\ldots ,\lambda_{ij}^{\left(M_{j}\right)}$.

(4) The coefficients obtained in the matrix $C_{ij}$ are used to generate a sparse rotation matrix $R_{i},$ which is given as follows:

(10) $$R_{i}=\left[\begin{array}{cccc} \lambda_{i1}^{\left(1\right)},\ldots ,\lambda_{i1}^{\left(M_{1}\right)} & \left\{0\right\} & \cdots & \left\{0\right\}\\ \left\{0\right\} & \lambda_{i2}^{\left(1\right)},\ldots ,\lambda_{i2}^{\left(M_{2}\right)} & \cdots & \left\{0\right\}\\ \vdots & \vdots & \ddots & \vdots \\ \left\{0\right\} & \left\{0\right\} & \cdots & \lambda_{iK}^{\left(1\right)},\ldots ,\lambda_{iK}^{\left(M_{K}\right)} \end{array}\right].$$

In the classification process, let $d_{ij}(xR_{i}^{\lambda })$ be the probability generated by the classifier $T_{i},$ which is used to determine whether x belongs to class $y_{i}.$ Next, the average combination method is used to calculate the confidence of each class in a given test sample, and the formula is as follows:

(11) $$\omega_{j}(x)=\frac{1}{L}\sum_{i=1}^{L}d_{ij}(xR_{i}^{\lambda }).$$

Finally, the test sample x will be assigned to the class with the greatest confidence.

## 4. Conclusions

In this paper, we proposed a computational method using only protein sequence information to predict PPIs. The proposed method can accurately predict the interaction among proteins by combining the local binary pattern algorithm and rotation forest classifier. In the experiment, we validated the proposed model on the *Yeast, Human*, and *H. pylori* datasets using the 5-fold cross-validation method. To evaluate the performance of the proposed model, we compared it with the SVM model and the existing methods in the same dataset. Among them, the proposed method obtained average prediction accuracy of 92.12%, 96.21%, and 86.59% on the *Yeast*, *Human*, and *H. pylori* datasets, respectively. Comparing these good experimental results, it can be seen that the proposed method is reliable and feasible for predicting PPIs. In addition, we also evaluate the proposed model in four independent datasets, including *C. elegans, H. pylori, H. sapiens*, and *M. musculus*. In the above experiments, the proposed models have achieved excellent results. This demonstrated that the proposed model is highly competitive and can be used as an effective tool for PPIs prediction. In future research, we will introduce a deep learning algorithm into the model to help the model achieve better prediction performance.

## Figures and Tables

**Table 1:** 5-fold cross-validation results obtained using the proposed method on three datasets.

Data Sets	ACC (%)	PE (%)	SN (%)	MCC (%)	AUC (%)
*Yeast*	92.12 ± 0.54	94.20 ± 0.78	89.76 ± 0.96	85.46 ± 0.92	96.11 ± 0.77
*Human*	96.21 ± 0.76	97.23 ± 1.19	94.77 ± 1.09	92.70 ± 1.42	98.62 ± 0.48
*H. pylori*	86.59 ± 0.48	87.70 ± 1.89	85.17 ± 2.20	76.73 ± 0.74	92.69 ± 0.48

ACC = accuracy, PE = precision, SN = sensitivity, MCC = Matthews correlation coefficient, AUC = Area Under the Curve.

**Table 2:** Comparison of the results of the proposed model and Support Vector Machine (SVM) model in three datasets.

Dataset	Classifier	ACC (%)	PE (%)	SN (%)	MCC (%)	AUC (%)
*Yeast*	RF	92.12 ± 0.54	94.20 ± 0.78	89.76 ± 0.96	85.46 ± 0.92	96.11 ± 0.77
	SVM	86.99 ± 0.43	88.05 ± 0.88	85.62 ± 1.23	77.36 ± 0.64	93.66 ± 0.64
*Human*	RF	96.21 ± 0.76	97.23 ± 1.19	94.77 ± 1.09	92.70 ± 1.42	98.62 ± 0.48
	SVM	92.56 ± 0.70	93.71 ± 1.06	90.47 ± 0.82	86.18 ± 1.23	97.36 ± 0.65
*H. pylori*	RF	86.59 ± 0.48	87.70 ± 1.89	85.17 ± 2.20	76.73 ± 0.74	92.69 ± 0.48
	SVM	81.62 ± 1.22	80.73 ± 3.79	83.40 ± 3.56	69.93 ± 1.56	89.52 ± 0.53

**Table 3:** Performance comparison of different methods on *Yeast* dataset.

Author	Model	ACC (%)	PE (%)	SN (%)	MCC (%)
Guos’ work [34]	ACC	89.33 ± 2.67	88.87 ± 6.16	89.93 ± 3.68	N/A
	AC	87.36 ± 1.38	87.82 ± 4.33	87.30 ± 4.68	N/A
You et al.’s work [17]	PCA-EELM	87.00 ± 0.29	87.59 ± 0.32	86.15 ± 0.43	77.36 ± 0.44
Yang et al.’s work [31]	Cod1	75.08 ± 1.13	74.75 ± 1.23	75.81 ± 1.20	N/A
	Cod2	80.04 ± 1.06	82.17 ± 1.35	76.77 ± 0.69	N/A
	Cod3	80.41 ± 0.47	81.86 ± 0.99	78.14 ± 0.90	N/A
	Cod4	86.15 ± 1.17	90.24 ± 1.34	81.03 ± 1.74	N/A
Zhou et al.’s work [32]	SVM + LD	88.56 ± 0.33	89.50 ± 0.60	87.37 ± 0.22	77.15 ± 0.68
Wang et al.’s work [36]	PCVM + ZM	94.48 ± 1.2	93.92 ± 2.4	95.13 ± 2.0	89.58 ± 2.2
Our method	SVM + PSSM	86.99 ± 0.43	88.05 ± 0.88	85.62 ± 1.23	77.36 ± 0.64
	RF + PSSM	92.12 ± 0.54	94.20 ± 0.78	89.76 ± 0.96	85.46 ± 0.92

ACC: Auto Cross Covariance; AC: Auto Covariance; PCA-EELM: Principal component analysis-ensemble extreme learning machine; LD: Local description; PCVM + ZM: Probabilistic Classification Vector Machines+ Zernike Moments.

**Table 4:** Performance comparison of different methods on *Human* dataset.

Model	ACC (%)	SN (%)	MCC (%)
LDA + RF [37]	96.4	94.2	92.8
LDA + RoF	95.7	97.6	91.8
LDA + SVM	90.7	89.7	81.3
AC + RF	95.5	94.0	91.4
AC + RoF	95.1	93.3	91.0
AC + SVM	89.3	94.0	79.2
Our method	96.21	94.77	92.70

LDA: Linear discriminant analysis; RoF: Rotation forest; RF: Random forest.

**Table 5:** Predicted results on four independent datasets.

Species	Test Pairs	ACC (%)
*C. elegans*	4013	94.82
*H. pylori*	1420	94.79
*H. sapiens*	1412	95.11
*M. musculus*	313	93.93

## Supplementary Materials

The following are available online at https://www.mdpi.com/1422-0067/20/14/3511/s1, Tables S1–S3.
