# Supplementary material for: An Ensemble Classifier to Predict Protein–Protein Interactions by Combining PSSM-based Evolutionary Information with Local Binary Pattern Model
Source: Int J Mol Sci. 2019 Jul 17;20(14):3511. doi: 10.3390/ijms20143511 (PMC6679202; doi:10.3390/ijms20143511)
Supplement: Supplementary file 1 [file ijms-20-03511-s001.pdf]

Table S1: 5-fold cross-validation results obtained by using the proposed method on *Yeast* dataset.

Testing set	ACC(%)	PE(%)	SN(%)	MCC(%)	AUC(%)
1	93.03	95.22	90.87	87.02	97.12
2	91.86	93.70	89.66	85.03	95.55
3	91.60	94.81	88.30	84.58	95.20
4	92.04	93.37	90.33	85.34	96.56
5	92.05	93.90	89.63	85.34	96.13
Average	92.12±0.54	94.20±0.78	89.76±0.96	85.46±0.92	96.11±0.77

Table S2: 5-fold cross-validation results obtained by using the proposed method on *Human* dataset.

Testing set	ACC(%)	PE(%)	SN(%)	MCC(%)	AUC(%)
1	96.20	98.54	93.56	92.67	98.64
2	97.43	98.04	96.53	94.97	99.33
3	95.71	96.62	94.42	91.77	98.52
4	95.47	95.54	94.52	91.27	97.99
5	96.26	97.41	94.83	92.79	98.61
Average	96.21±0.76	97.23±1.19	94.77±1.09	92.70±1.42	98.62±0.48

Table S3: 5-fold cross-validation results obtained by using the proposed method on *H. pylori* dataset.

Testing set	ACC(%)	PE(%)	SN(%)	MCC(%)	AUC(%)
1	86.28	89.14	82.35	76.24	93.19
2	85.93	90.20	83.44	75.71	92.22
3	87.14	86.90	87.20	77.58	93.11
4	86.79	85.66	87.19	77.05	92.18
5	86.82	86.59	85.66	77.05	92.74
Average	86.59±0.48	87.70±1.89	85.17±2.20	76.73±0.74	92.69±0.48
